# Influence of Ultrasonic Wind Sensor Position on Measurement Accuracy under Full-Scale Conditions

**DOI:** 10.3390/s20195640

**Published:** 2020-10-02

**Authors:** Tomasz Lipecki, Paulina Jamińska-Gadomska, Andrzej Sumorek

**Affiliations:** Department of Structural Mechanics, Faculty of Civil Engineering and Architecture, Lublin University of Technology, Nadbystrzycka 40, 20-618 Lublin, Poland; p.jaminska@pollub.pl (P.J.-G.); a.sumorek@pollub.pl (A.S.)

**Keywords:** wind engineering, wind speed, full-scale measurements, wind tunnel, sonic anemometer

## Abstract

A system designed for making field measurements of wind action on engineering structures is described. The system is composed of sonic anemometers, differential pressure sensors, a barometer, and a thermohygrometer. The focus of this study is to determine the indications of sonic anemometers; to accomplish this goal, wind tunnel tests were performed. The tests did not involve checking the accuracy of the devices themselves, but determining their indications under field measurement conditions where certain unavoidable errors resulting from their installation can appear. The anemometer measurement uncertainty with respect to wind speed and angle was determined. The devices were rotated in a horizontal plane and inclined against and with the mean wind speed direction in a wind tunnel. Different tunnel wind speeds were tested. The results indicate stable device readings at different horizontal plane positions at different wind speeds and a low sensitivity to changes in inclination against the inflow.

## 1. Introduction

Environmental engineering, especially wind engineering, uses three kinds of experiments: in situ measurements, model tests in climatic chambers or wind tunnels, and computational fluid dynamics (CFD) simulations. The last approach must still be validated by in situ or model measurements. Full-scale data collection does not need to use similarity criteria unlike wind tunnel or CFD simulation approaches. However, measuring climate parameters, including wind speed and direction, at full scale is quite challenging, especially under construction site conditions. To carry out such measurements, commercially available devices are used, or an adequate measuring system is built on the basis of these. An up-to-date, comprehensive literature review of cup, sonic, and pressure anemometers, lidar research aircrafts, and unmanned aircraft systems (UAS) techniques for measuring wind gusts in environmental engineering was presented in [1]. Various kinds of anemometers, differential pressure sensors, lidar, etc. are employed to study the effects of wind [2,3,4], whereas accelerometers or global positioning system (GPS) devices [5,6,7] are often necessary in dynamic tests of civil engineering structures. These types of measuring devices or systems are typically used to monitor the behavior of existing structures and are used much less frequently in the design stage. The accuracy of sonic anemometers and different coordinate rotation methods for posterior correction of measurement data in practical applications was presented in [8,9].

Recently, extensive results were made available regarding monitoring of the wind-induced dynamic responses of high-rise buildings under typhoon conditions in China (e.g., [10,11,12,13]). Measurements focused on pressure changes on the roofs of low-rise buildings during typhoons were presented in [14,15]. Recently, wind pressures were measured at full scale on rooftop photovoltaic arrays [16], different shaped roofs [17], low-slope membrane roofs [18], and on walls of medium-rise buildings [19].

Other full-scale experiments conducted using anemometers and pressure taps focused on issues related to roof ventilation [20], double-skin building façades [21], urban array of buildings [22], or vehicular tunnels [23].

Full-scale experiments that focused on wind speed and direction involved measurements of wind flow between arrays of solar photovoltaic panels with respect to dust deposition on and between them [24] or measurements of crosswinds acting on moving highway vehicles [25]. Data from sonic anemometers were also used to determine the relationship between strong winds and rainfalls in tropical climate and their coupled influence on building structures [26].

Roof and wall wind pressure, and the flow characteristics were thoroughly measured at full scale near a low-rise building at Texas Tech [27,28], and especially around an experimental 6 × 6 × 6 m^3^ Silsoe cube [29,30]. The data gathered throughout the years from the experiments on these two structures are used to validate wind tunnel model tests and CFD simulations.

Most of the works presented above concerned issues related to civil engineering and to the estimation of the wind load acting on the structures using measurements. Other experiments concerned wind speed measurements performed in areas intended for wind farms or monitoring of large and micro wind turbines (e.g., [31,32]).

Much research is still aimed at the precise definition of the vertical profiles of the mean wind speed and turbulence in flat open areas, as well as in city centers [33,34,35,36,37,38]. In addition to anemometers and lidar, small aircraft systems or quadcopters are used in this type of research. The use of unmanned aircraft systems, including both airplanes and drones, gives new possibilities in measuring wind speed and direction and other environmental parameters. Tests of various types of UAS and their practical use for measuring wind speed and direction were presented, among others, in [39,40,41].

To sum up, most previous studies focused on structural monitoring or determining the vertical changes in the wind characteristics. In many of the examples mentioned above, measurements of wind speed and direction or wind pressure were often accompanied by measurements of other environmental parameters, such as air temperature and humidity, atmospheric pressure, or insolation. There are few examples of measuring devices used to determine the temporary wind load on engineering structures. Of course, long-term measurements provide much more reliable data; however, economic considerations, such as sensor cost and the necessity to leave them for a long time in one location, or the need for a quick determination of the wind impact during the design process, often require the use of portable systems that can be easily set up under given conditions for shorter periods of time. This paper presents a system designed for measuring the wind load on structures and the flow parameters around them. The system also provides simultaneous recordings of other environmental parameters, such as atmospheric pressure, air humidity, and temperature. The system requirements and the final solution (including the sensors used) are presented. A practical application in full-scale testing is demonstrated by wind speed and direction measurements on façade scaffoldings. Doubts related to the correctness of anemometer indications associated with the possible inaccuracy of their installation under construction site conditions led to a controlled experiment carried out in a boundary-layer wind tunnel. Sensitivity analyses of the results to possible inaccuracies in sensor assembly or rapid changes in flow speed are presented.

## 2. Measuring System

### 2.1. System Requirements

The main application of the designed system is assumed to be in situ measurements of wind speed and direction, as well as wind speed differential pressure. In addition, the system had to meet the criterion of mobility, as well as enable fast and easy installation under construction site conditions. The system was prepared as part of a research project entitled “*Modeling of risk assessment of construction disasters, accidents, and dangerous incidents at workplaces using scaffoldings*”, which was focused on ensuring the safety of employees working on scaffoldings. Considering the construction site operating conditions, the system had to be resistant to dust, rainfall, vibrations etc. Taking into account all the boundary conditions, the following initial assumptions were made: (1) system mobility allows for safe and repeated transport; (2) wind speed measurements must be made up to 60 m/s in two and, if possible, three directions; (3) the system must measure wind speed differential pressure; (4) the system must measure atmospheric pressure, air humidity, and temperature; (5) the sensor accuracy must be in the range of 1–2%; (6) the maximum distance between the measuring points and the data acquisition point is 30 m; (7) the minimum sampling frequency is equal or greater than 20 samples per second for each channel (this value refers to the sampling rate of the air velocity obtained from the sensor by the electronic measurement system; it was assumed that the electronic measurement system must sample the value from the flow sensor with a frequency at least twice as high as the frequency of changes of the signal from the sensor); (8) the system must be able to carry out measurements at a construction site; (9) the system should have the potential for future development, e.g., by adding wireless communication or by increasing the number of measuring points.

### 2.2. System Elements

**Figure 1:**
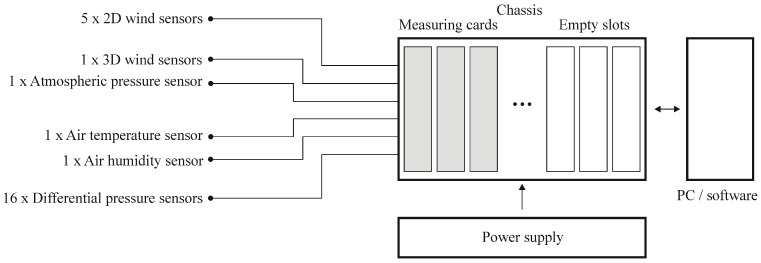
Measuring system configuration.

**Figure 2:**
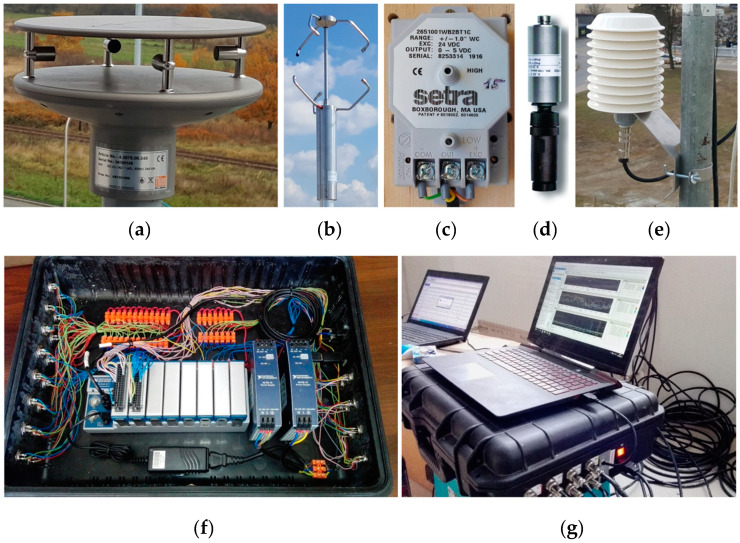
System components: (**a**) 2D anemometer; (**b**) 3D anemometer; (**c**) pressure sensor; (**d**) barometer; (**e**) thermohygrometer; (**f**,**g**) central unit.

The compromise between measurement requirements and cost determined the final system configuration, which is shown in Figure 1. Sensors commonly used in meteorological stations were implemented to measure wind speed and direction and other atmospheric parameters. The sensors had high accuracy and high sampling frequency and are resistant to weather conditions. The system consists of one Thies Clima Ultrasonic Compact three-dimensional (3D) anemometer and five Thies Clima Ultrasonic Compact two-dimensional (2D) anemometers with basic characteristics, as listed in Table 1. The 2D anemometers allow two-dimensional acquisition of the horizontal wind speed components, whereas the 3D device measures three wind speed components. In addition, the system uses the Ammonit AB60 barometer and the Ammonit S52100 thermohygrometer with specifications shown in Table 2. Finally, 16 Setra differential pressure sensors were adopted in the system—eight with 60 Pa pressure range and the other eight with 250 Pa pressure range, values which correspond to wind speeds of approximately 10 m/s and 20 m/s. The sensor accuracy was 1%, and the maximum overpressure was 69 kPa. The central unit for data recording was powered by two NI PS-15 power supplies. Two (out of eight possible) NI 9205 32-input measurement cards placed in NI CompactDAQ 8-USB chassis slots are currently used. All components were installed in a durable and damage-resistant case, which enables safe transport of the system. National Instruments Signal Express software ver. 15.0.0 manages the system’s adaptation to the planned tasks. The options of control signal channel settings, signal parameters, recording, etc. are maintained. The acquired data handling must be performed by external software on the basis of the saved .csv files. The system sensors and the central unit are shown in Figure 2.

### 2.3. System Application in Civil Engineering Practice

**Figure 3:**
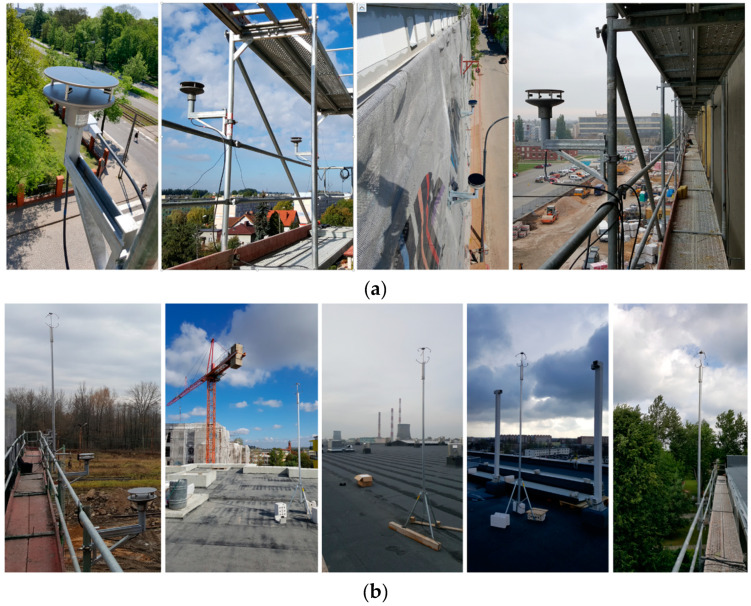
System field application: (**a**) 2D anemometers; (**b**) 3D anemometers.

The created system was used in practice. An example of measurements conducted with 2D anemometers mounted in the contraction formed by two converging walls of a bell tower was described in detail in [42]. In these experiments, three 2D anemometers measured wind speeds and directions at the front, back, and middle of the passage. The main purpose was to determine the wind speed amplification or reduction factor caused by the cross-section contraction and to validate CFD simulations. The system was also used many times during a project focused on scaffolding worker safety, as mentioned in Section 2.1. Measurements were made on 36 façade scaffoldings. Five 2D anemometers measured wind speeds and directions at a distance of 0.36 m in front of the external surface of the scaffolding, while the 3D anemometer measured the reference speed and direction at the construction site. The 2D anemometers were mounted to the stands of the scaffoldings with the use of consoles at the penultimate deck level (Figure 3a). The location of the 3D device was chosen to ensure the most undisturbed flow, i.e., on a mast mounted on the roof of the building or to the highest stand (Figure 3b). The atmospheric pressure, air temperature, and humidity were also measured. An example of measurements on scaffolding structure was presented in [43].

**Figure 4:**
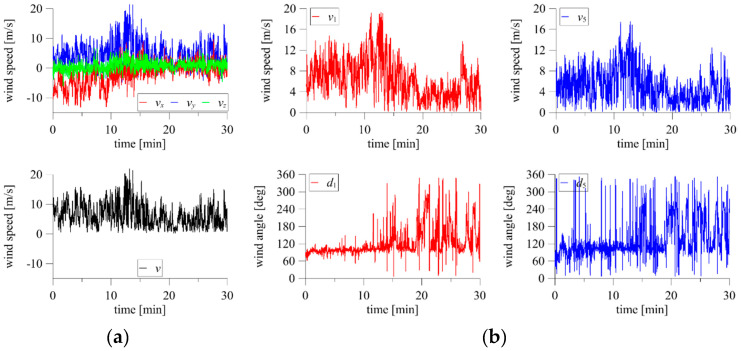
Full-scale measurements of wind speed and direction using: (**a**) 3D anemometer; (**b**) two 2D anemometers.

**Figure 5:**
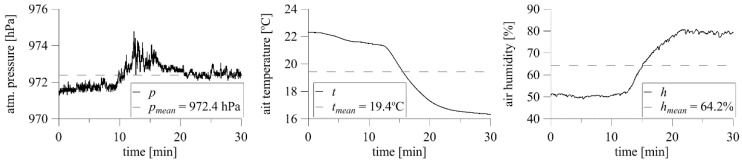
Full-scale measurements of atmospheric pressure, air temperature, and humidity.

An example of test results from a façade scaffolding standing against a 30 m high apartment building is shown in Figure 4. The results include 30 min time series of three wind speed components (*v_x_*, *v_y_*, and *v_z_*) and the resultant wind speed *v*, measured on the building’s roof with a 3D anemometer (Figure 4a), and two examples of wind speed, *v*_1_ and *v*_5_, and direction, *d*_1_ and *d*_2_, measured in front of the scaffolding with 2D anemometers (Figure 4b). The significant wind angle fluctuations visible in Figure 4b are caused by the way of data recording by the system. The angles 0° and 360° denote the same flow angle, which was perpendicular to the building façade in this research. The system recorded small fluctuations around this direction as greater than 0° or less than 360°. The 2D devices were placed approximately 28 m above the ground. A wind angle equal to 0° indicates that the wind action was perpendicular to the scaffolding plane. The two 2D anemometers referenced in the plots were spaced approximately 50 m apart in the horizontal direction; however, the wind characteristics are clearly similar, especially in terms of wind speed. In addition, 30 min time histories of atmospheric pressure, air temperature, and humidity were measured simultaneously, and their mean values are presented in Figure 5. The short-duration pressure fluctuations visible on the plots are insignificant, and the plots show a clear decrease in temperature and an increase in humidity caused by the passing storm.

## 3. Wind Tunnel Tests

### 3.1. Wind Tunnel Set-Up

During many full-scale tests carried out mainly on scaffolding, it was often difficult or impossible to accurately control the installation of anemometers under field conditions, especially to provide high-accuracy leveling and angle settings. Therefore, the 2D and 3D anemometers were tested in a wind tunnel to determine acceptable levels of additional errors not directly related to the device precision, but to possible operational inaccuracies that can occur during the assembly process. In other words, what margin of nominal position deviation can be maintained in the exact positioning of the equipment at the construction site, and how do these errors affect the result accuracy? In addition, the device’s reading stability at different speeds and wind flow angles was determined.

The measurements were carried out in the wind tunnel at the Warsaw University of Technology Power in the Aeronautical Engineering Aerodynamics department. The wind tunnel was a closed-loop type with two test sections: a larger section for environmental and aerodynamic studies and a smaller section for aeronautical research. The environmental section was used in the experiment. The dimensions of the test section were 2.5 m width, 2.1 m height, and 10 m length. All roughness elements were removed from the test section, and a uniform flow was produced. During the experiment, the wind speed was controlled by measuring instruments permanently located in the wind tunnel. The maximum measured wind speed value was 22 m/s, and the turbulence intensity at the height of the tested anemometers was equal to 5% at every speed level. The temperature was 28 °C, the atmospheric pressure was 990 hPa, and the air density was 1.146 kg/m^3^.

**Figure 6:**
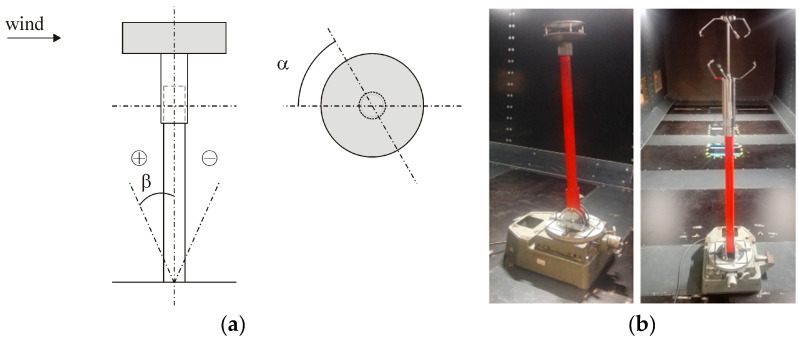
Anemometer settings: (**a**) scheme with definition of *α* and *β*; (**b**) 2D and 3D devices in the wind tunnel.

The anemometers were attached to a pipe mounted to the base placed in the tunnel floor, which enabled rotation around the vertical and horizontal axes of the tunnel. In the basic position, the anemometer’s measuring elements were located approximately 1 m above the tunnel floor. The assembly configuration and the definition of *α* and *β* angles are shown in Figure 6. Measurements were taken for three arrangements:

1. The anemometer was rotated in the horizontal plane at the following *α* angles: 0° (360°), 45°, 90°, 135°, 180°, 225°, 270°, and 315°. The angle of inclination in the vertical plane was constant, *β* = 0°. The tests were performed for one 3D and five 2D anemometers. The tunnel wind speed was constant.
2. The anemometer was rotated in the vertical plane at the following *β* angles: −15°, −10°, −5°, 0°, +5°, +10°, +15°, +20°, +25°, +35°. The anemometer inclination in the direction against the flow was assumed as positive and that with the flow was assumed as negative. The tests were performed for one 2D and one 3D anemometer in two horizontal positions, at *α* = 0° and *α* = 90°. The tunnel wind speed was constant.
3. The horizontal and vertical positions were constant, *α* = 0° and *β* = 0°. The tunnel wind speed was changed gradually. The tests were performed for one 2D and one 3D anemometer.

### 3.2. Influence of Horizontal Rotation Angle (Changing α, β = 0°)

Tests were performed for each of five 2D anemometers in the full range of wind flow angle *α* from 0° to 315°, every 45°. Anemometers were initially placed at *α* = 0° and then rotated along the vertical axis, without changing any wind tunnel flow parameters. These initial tests were conducted to check whether the accuracy of the anemometers’ indication was at the same level at different arrangement angles against the tunnel inflow.

**Figure 7:**
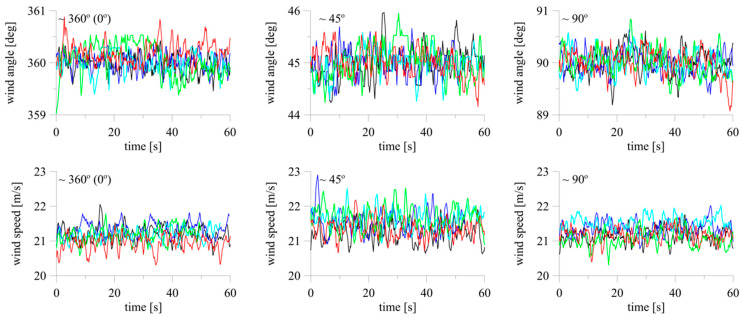
Fluctuations of wind speed and angle for 2D anemometers. Colors differentiate anemometers A1–A5.

All anemometers showed similar levels of wind angle fluctuations with time, with a maximum of approximately 1°, which corresponds to 2.2% of the mean angle. In the majority of cases, fluctuation levels were very low, at 0.2–0.4% of the mean angle. The maximum time fluctuation of the wind speed was 1.35 m/s against the mean and was measured on one device at *α* = 45°, which corresponds to 6.27% of the mean speed. For the remaining anemometers, the maximum fluctuations ranged from 0.4–0.7 m/s, which corresponds to 2–4% of the mean speed (Figure 7).

**Figure 8:**
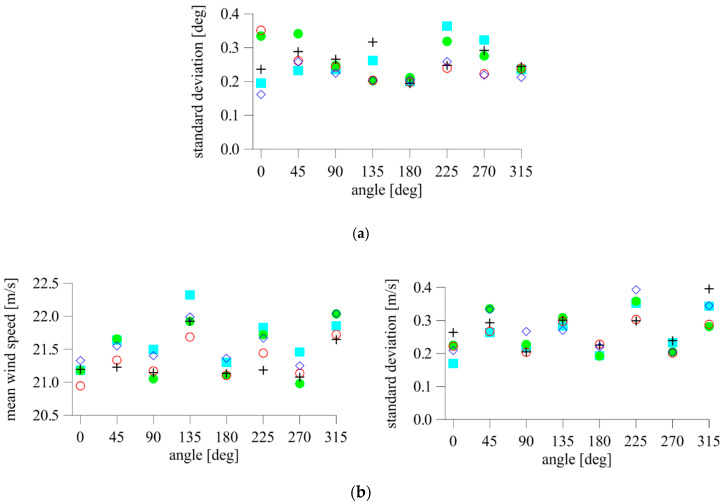
Mean and standard deviations of (**a**) wind angle and (**b**) wind speed; +—A1, ◇—A2, ■—A3, ●—A4, ○—A5.

To quantitatively compare the measurements, the mean wind speed and standard deviations of the wind speed and angle were calculated for each setting and device. The mean wind speed for all values of *α* and anemometers ranged from 20.95–22.33 m/s (see Figure 8b). Taking the mean wind speed of each device at *α* = 0° as the reference, the maximum obtained differences were 0.73 m/s (*α* =135°), 0.71 m/s (*α* = 315°), 1.14 m/s (*α* = 135°), 0.85 m/s (*α* = 315°), and 0.78 m/s (*α* = 315°) for the 2D anemometers. The corresponding percentage differences were 3.42%, 3.34%, 5.37%, 4.03%, and 3.73%, respectively.

The ranges of values of standard deviations of the angle and speed for subsequent anemometers were equal to 0.19–0.32°, 0.16–0.26°, 0.20–0.36°, 0.20–0.34°, and 0.20–0.35° (Figure 8a), and 0.21–0.40 m/s, 0.21–0.39 m/s, 0.17–0.35 m/s, 0.19–0.36 m/s, and 0.20–0.30 m/s, respectively (Figure 8b). None of the devices indicated results that deviated significantly. The standard deviation had higher values at *α* = 45°, 135°, 225°, and 315°, especially in relation to the wind speed. It seems that the reason is the construction of the compact sonic anemometer. The upper and lower parts of the device are connected by four brackets on which the sensors are mounted (Figure 2a). One of the brackets is windward in these positions. Vortices shed from its surface introduce additional air flow disturbances between the opposite brackets, which influence the determination of speed in this direction.

**Figure 9:**
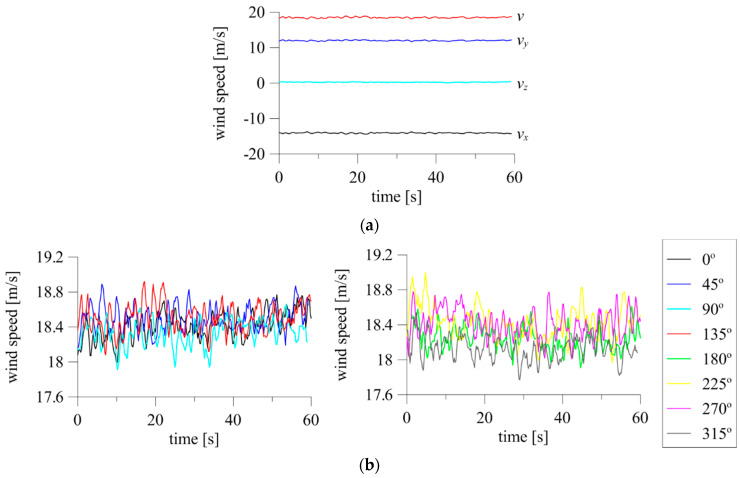
Fluctuations of wind speed: (**a**) components and resultant, for *α* = 135°; (**b**) resultant for all angles.

Figure 9 shows examples of time fluctuations of the resultant wind speed, *v*, and its components, *v_x_*, *v_y_*, *v_z_* for *α* = 135°, as well as resultant speed curves for all *α* measured with the 3D anemometer. Time fluctuations of the resultant speed around the mean value did not exceed 0.57 m/s, which is 3.08% (*α* = 225°), and, for the majority of the angles, it was approximately 2%. The maximum time fluctuations of the angle were equal to 0.88° and were obtained at *α* = 45°. Taking, as previously, the mean wind speed measured at *α* = 0° as the reference, the maximum difference from this value was obtained at *α* = 315° and was equal to 0.32 m/s, which is 1.73% of the mean value.

**Figure 10:**
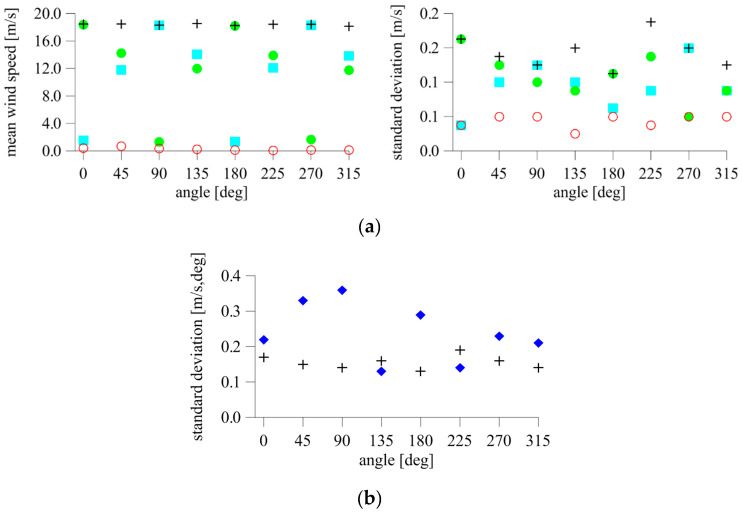
Mean and standard deviation of (**a**) components and resultant wind speed, ■—*v_x_*, ●—*v_y_*, ○—*v_z_*, +—*v*, and (**b**) resultant wind speed and angle, +—speed, ◆—angle.

The mean values of wind speed components and standard deviations of the angle and resultant speed are presented in Figure 10. In each case, the value of the vertical velocity component, *v_z_*, was close to zero, which indicated laminar flow in the tunnel in the vertical section. The highest standard deviation of resultant speed was calculated at *α* = 225° and was equal to 0.19 m/s, whereas the highest standard deviations of both horizontal components, *v_x_* and *v_y_*, were 0.16 m/s (*α* = 270°) and 0.17 m/s (*α* = 0°), respectively. The standard deviation of the resultant speed was determined by the value of the predominant component at the given angle, which means that, for a lower component value, its standard deviation was lower as well. Relatively higher standard deviations (compared to wind speed) were obtained for angles. The highest was 0.36° (*α* = 90°), and the lowest were 0.13° (*α* = 135°) and 0.14° (*α* = 225°). This finding was contrary to the 2D anemometers’ indications, but was caused by the different construction of the 3D sensor (see Figure 2b). For most settings except *α* = 135° and *α* = 225°, the standard deviations of the angle were much higher than those of the speed (Figure 10b).

### 3.3. Influence of Inclination (Changing β, α = 0° and α = 90°)

**Figure 11:**
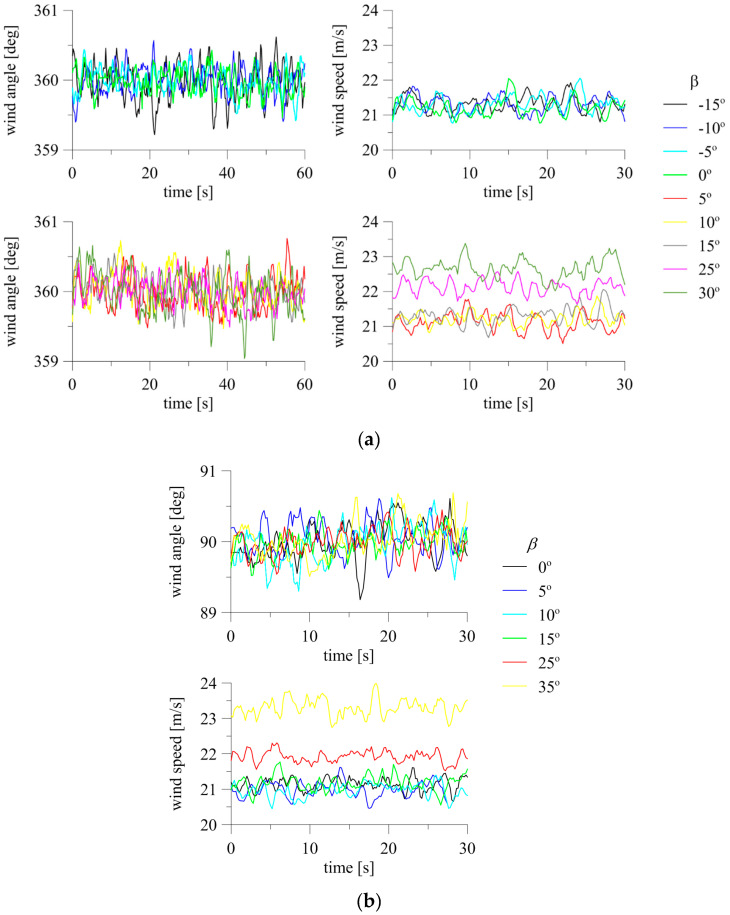
Fluctuations of wind angle and speed at changing *β* for 2D anemometer: (**a**) *α* = 0°; (**b**) *α* = 90°.

The effect of inclination of the device in the direction of or opposite to the air stream in the tunnel was examined. When using the system at construction sites, determining the level of change in measurement results due to possible assembly inaccuracy is very important. To investigate this issue, the 2D anemometer was installed in the horizontal plane at *α* = 0°. The inclination described by *β* was changed gradually, from −15° to +30° (see Figure 6a). The tests were repeated at *α* = 90° just for the positive inclination at *β* = 0° to +35°. Indications of the 3D anemometer were only checked at *α* = 0° and at *β* = −15° to +15°. Time changes of wind angle and speed measured with the 2D anemometer for *α* = 0° and *α* = 90° are presented in Figure 11. In the case of *α* = 0°, the graphs were divided into two parts, *β* ≤ 0° and *β* > 0°, to increase their readability. The reference wind speed measured at the initial position at *β* = 0° was 21.2 m/s for *α* = 0° and 21.16 m/s for *α* = 90°.

**Figure 12:**
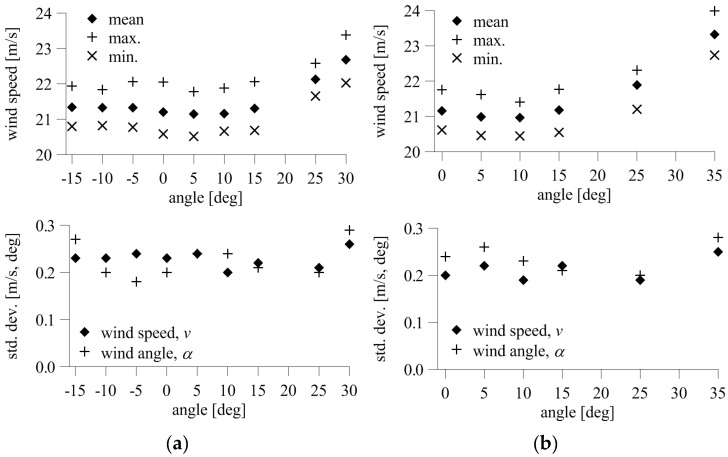
Mean and extreme wind speeds and standard deviations of wind speed and angle: (**a**) *α* = 0°; (**b**) *α* = 90°.

The indications of the device at *α* = 0° were similar for *β* between −15° and +15°, and significantly abnormal for higher inclinations, *β* = +25°, +30°. This result was confirmed by the mean and extreme values of speed presented in Figure 12. The mean speed differed from the reference value maximally by 0.13 m/s at *β* = +15°, which was 0.62% of the reference, and maintained the same level in the angle range *β* = −15° to +15°. The differences were much higher at *β* = +25°, +30°, equal to 0.92 and 1.48 m/s, or 4.36% and 6.96% of the reference value, respectively. The standard deviation of speed ranged from 0.20–0.24 m/s and was at the same level for *β* = −15° to +25°. The standard deviation of speed was only slightly higher, equal to 0.26 m/s, at *β* = +35°. The standard deviation of the angle was also at a similar level, equal to 0.18−0.27° at *β* = −15° to +25°, and 0.29° at *β* = +35°.

The mean speeds at *α* = 90° and at *β* = 0° to +15° were similar to each other and differed from the initial speed maximally by 0.20 m/s, which is 0.96% of the reference. The difference was still acceptable at *β* = +25°, and significantly deviated at *β* = +35°. The values were 0.73 and 2.16 m/s, which represent 3.44% and 10.20%, respectively. The standard deviations of speed and angle ranged from 0.19–0.22 m/s and 0.20–0.26°. The values were similar at *β* = 0° to +25°, and only slightly higher at *β* = +35°, for which they were 0.25 m/s and 0.28°.

The standard deviation was used as an indicator of the measurement uncertainty. The calculated values indicated a low influence of the vertical deviation in the 2D anemometer position on the measurement results. Inaccuracies could have a bigger impact on mean indications, which are nevertheless at a fairly low and acceptable level. The vertical inclination of the device at *β* = −15° to +15° did not seriously influence the indications.

**Figure 13:**
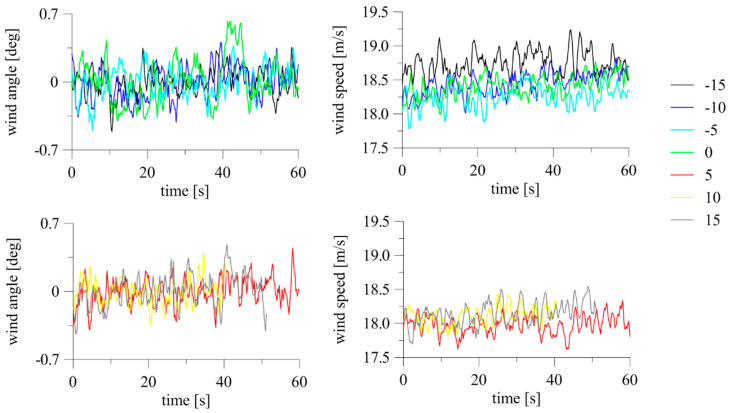
Fluctuations of wind angle and speed at changing *β* for 3D anemometer: *α* = 0°.

The time fluctuations of wind speed and angle at *β* = −15° to +15° for the 3D anemometer, divided into negative and positive ranges, are presented in Figure 13. The time fluctuations of the measured angle were insignificant, reaching a maximum of 0.6° and 0.5 m/s from the mean. There was a characteristic decrease in speed values corresponding to the increasing inclination of the anemometer against the wind direction (*β* > 0°). The reference wind speed, measured for initial position, was equal to 18.43 m/s. The maximum difference between the mean wind speed and the reference speed was 0.45 m/s at *β* = +5°, or 2.42% of the reference. For larger inclinations, the differences were slightly lower, at the level of 1–2%.

**Figure 14:**
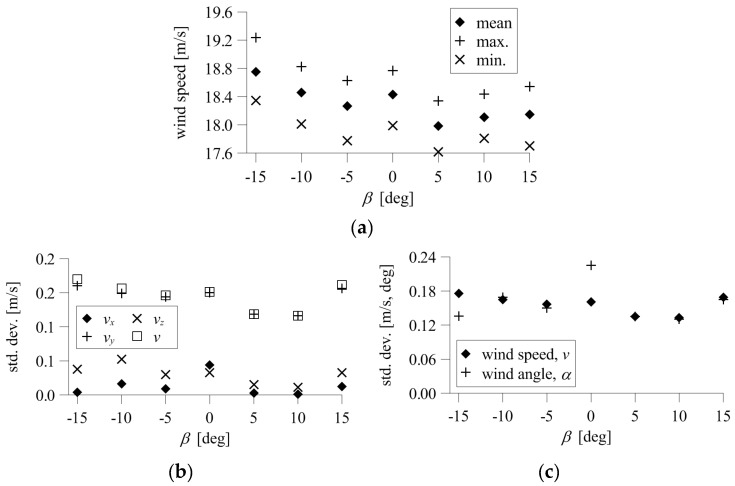
Results for the 3D anemometer: (**a**) mean and extreme resultant speed; (**b**) standard deviation of resultant speed, *v*, and components, *v_x_*, *v_y_*, *v_z_*; (**c**) standard deviation of resultant speed and angle.

Changes in the mean and extreme values of resultant speed and its components and standard deviations of the resultant speed and angle are shown in Figure 14. The standard deviations of speed for different *β* ranged from 0.13–0.18 m/s ere wwas mainly dependent on the *v_y_* component, which was directed along the flow in the tunnel for *α* = 0°, thus having a dominating value. The maximum standard deviation was calculated at *β* = −15° and *β* = +15, but was quite similar to values measured at smaller inclinations. Slightly lower values were received for *β* > 0°. The standard deviation of wind angle ranged from 0.13–0.23° and was the highest at the initial position at *β* = 0°.

The values of the standard deviation, which is an indicator of measurement uncertainty, show that, apart from exceptional settings, changing the inclination of the 3D sensor in the range of *β* from −15° to +15° introduces (similar to 2D devices) maximum speed uncertainty in the range declared by the device manufacturer.

### 3.4. Influence of Wind Speed (α = 0°, β = 0°)

**Figure 15:**
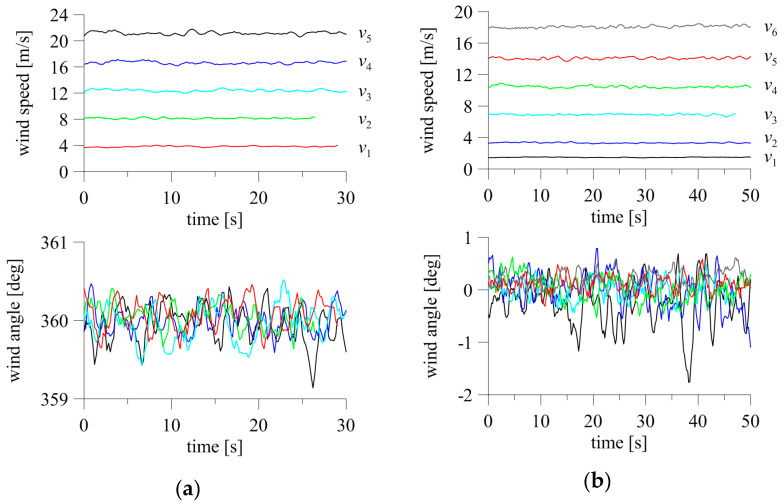
Fluctuations of wind angle and speed at changing flow speed for (**a**) 2D anemometer and (**b**) 3D anemometer.

The stability of the 2D and 3D anemometer indications was also checked against increasing wind speed in the tunnel. The anemometers were positioned at *α* = 0° and *β* = 0°. Five speeds were measured for the 2D anemometer and six for the 3D anemometer. The time fluctuations of speed and angle are shown in Figure 15. The maximum angle changes were within ±0.5° for all speed levels and both types of anemometers, except the lowest speeds, for which the fluctuations exceeded ±1° (3D device).

**Figure 16:**
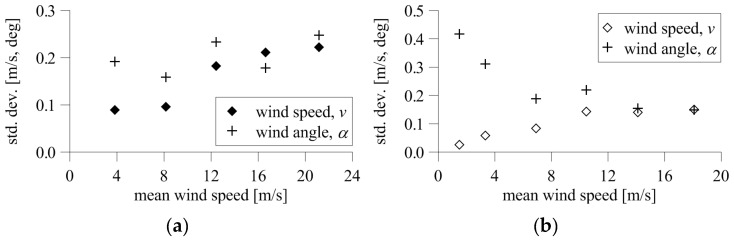
Standard deviation of wind speed and angle at changing flow speed for (**a**) 2D anemometer and (**b**) 3D anemometer.

The standard deviation of speed with a 2D anemometer was 0.09 m/s for the lowest speed, and increased with speed to a maximum of 0.22 m/s for the highest speed (Figure 16a). The standard deviation of angle was 0.16–0.25°, and no clear trend connected with speed levels was observed.

The standard deviation of the resultant speed of the 3D anemometer was 0.03 m/s for the lowest speed, and increased to 0.15 m/s for the highest speed. The opposite dependence occurred for wind angle, for which the standard deviation at the lowest speed was the highest and equal to 0.42°, decreasing quite clearly with increasing wind speed to 0.15° for the highest speed (Figure 16b).

## 4. Conclusions

This paper described a portable system for measuring environmental parameters, mainly wind direction and speed, under construction site conditions. An example of a full-scale experiment was given. The influence of the anemometers’ mounting inaccuracies on the correctness and stability of the wind speed and angle measurements was determined in tests performed under controlled conditions in a wind tunnel.

The measurement uncertainty estimated on the basis of standard deviation was calculated. For examined angles *α* (anemometer rotation in the horizontal plane), it reached maximum values of 0.36° and 0.40 m/s for the 2D devices, while, for the 3D device, maximum values were 0.36° and 0.19 m/s for angle and wind speed, accordingly. For all *α* angles, the measurement results were within the manufacturer’s accuracy limits (see Table 1), equal to 2° and approximately 0.4 m/s (2% of the mean value) for the 2D anemometer, and 1° and approximately 0.19 m/s (1% of the mean value) for the 3D version. The sensor readings were acceptable and stable at every horizontal position. The differences in the results indicate that the 2D devices readings may vary slightly depending on the angle, showing disturbances caused by their structural elements. This finding was not observed for the 3D anemometer, which was generally less prone to changes in vertical and horizontal angles.

The measurement uncertainty estimated during tests for various angles *β* = −15° to +15°, determining the anemometer position in the vertical plane, reached the maximum values of 0.27° and 0.24 m/s for the 2D anemometer, and 0.23° and 0.18 m/s for the 3D version. The changes in the mean wind speed in relation to the reference were also acceptable. The 2D anemometer indications were similar at *α* = 0° and *α* = 90°.

The measurement uncertainty estimated during tests for *α* = 0° and *β* = 0° and different levels of wind speed in the tunnel reached maximum values of 0.25° and 0.22 m/s for the 2D devices and 0.42° and 0.15 m/s for the 3D device. The standard deviation of the speed increased with increasing tunnel flow speed for both types of anemometers. On the other hand, the standard deviation of the angle decreased as the flow speed increased, at least for the 3D anemometer.

The general conclusion according to wind tunnel tests of sonic anemometers, both 2D and 3D, is that they are not very sensitive to possible inaccuracies which can appear due to their assembly under field conditions. The measured levels of angle and wind speed deviations for all devices were acceptable in field measurements for which the system was intended and designed. As mentioned in Section 2.3, the system has been used many times to determine the wind effects on engineering structures, mainly scaffolding. In the near future, research works are planned related to, inter alia, the measurement of wind speed and pressure on the double façades of buildings, and the impact of acoustic barriers at expressways on wind conditions on the road.

## Figures and Tables

**Table 1:** Basic anemometer specifications. 2D, two-dimensional; 3D, three-dimensional.

Specification	3D	2D
Wind speed range	0.01–85 m/s	0.01–75 m/s
Maximum resolution of wind speed	0.01 m/s	0.01 m/s
Accuracy of wind speed	±0.1 m/s (*v* < 5 m/s) ±1% (5 < *v* < 35 m/s) ±2% (35 < *v* < 85 m/s)	±0.2 m/s (*v* < 5 m/s) ±2% (5 < *v* < 60 m/s)
Wind speed direction range	360°	360°
Maximum resolution of wind speed direction	0.1°	0.1°
Accuracy of wind speed direction	±1° (1 < *v* < 35 m/s), ±2° (35 < *v* < 65 m/s), ±4° (65 < *v* < 85 m/s)	±2° (*v* > 1 m/s)

**Table 2:** Basic barometer and thermohygrometer specifications.

Specification	Barometer	Thermohygrometer
Humidity/atmospheric pressure/temperature range	800–1100 hPa	−30 to +70 °C 0–100% RH
Accuracy	±1%	±0.2 °C (temperature) ±2% (humidity)
Working temperature range	−40 to +85 °C	−40 to +80 °C
